# Increased Chemokine Production is a Hallmark of Rhesus Macaque Natural Killer Cells Mediating Robust Anti-HIV Envelope-Specific Antibody-Dependent Cell-Mediated Cytotoxicity

**DOI:** 10.20411/pai.v10i1.734

**Published:** 2025-01-23

**Authors:** Junsuke Nohara, Tyler Evangelous, Madison Berry, Whitney Beck, Sarah Mudrak, Shalini Jha, R. Keith Reeves, Kevin J. Wiehe, Justin Pollara, Georgia D. Tomaras, Todd Bradley, Guido Ferrari

**Affiliations:** 1 Department of Molecular Genetics and Microbiology, Duke University School of Medicine, Durham, NC; 2 Human Vaccine Institute, Duke University School of Medicine, Durham, NC; 3 Department of Surgery, Duke University School of Medicine, Durham, NC; 4 Duke Research and Discovery at RTP, Duke University Health System, Durham, NC; 5 Center for Human Systems Immunology, Duke University, Durham, NC; 6 Department of Pathology, Duke University School of Medicine, Durham, NC; 7 Genomic Medicine Center, Children's Mercy Research Institute, Children's Mercy Kansas City, Kansas City, MO; 8 Departments of Pediatrics and Pathology and Laboratory Medicine, University of Kansas Medical Center, Kansas City, MO; 9 Department of Pediatrics, UMKC School of Medicine, Kansas City, MO

**Keywords:** Rhesus Macaque, NK cells, Antibody-Dependent Cell Cytotoxicity, Single-Cell Gene Expression Analysis, Chemokine

## Abstract

**Background:**

Antibody-dependent cell-mediated cytotoxic (ADCC) response mediated by natural killer (NK) cells correlates with decreased infection risk in studies involving simian immunodeficiency virus (SIV)/simian-human immunodeficiency virus (SHIV), and human immunodeficiency virus (HIV) vaccine candidates. Currently, the heterogeneities of the functional subset of rhesus macaque natural killer (RMNK) cells are under-characterized.

**Method:**

We engaged the RMNK cells with ADCC-mediating anti-HIV-1 monoclonal antibodies (ADCCAbs) or anti-CD16 antibodies and used CD107a expression as the surrogate marker for RMNK cells actively involved in ADCC. CD107a^+^ and CD107a^–^ populations were analyzed individually using single-cell RNA sequencing.

**Results:**

Subsets of CD107a^+^ RMNK cells produced more chemokines than the others, suggesting that these cells not only eliminate infected cells but also provide immunoregulatory signals and potentially curb HIV-1 replication. Crosslinking of Fc gamma receptor IIIa via anti-CD16 antibodies resulted in a significantly higher percentage of degranulating cells than via ADCCAbs. However, the magnitude of degranulation and chemokine production was reduced by 6- to 30-fold.

**Conclusion:**

The quality and quantity of receptor engagement are important determinants of achieving an optimal level of the RMNK response.

## INTRODUCTION

Natural killer (NK) cells are a subset of lymphocytes that provide a first line of defense in eliminating infected and neoplastic cells [1]. NK cells recognize target cells through a wide repertoire of activating and inhibitory receptors, whose cumulative signals control the activation of the NK cells [2–5]. Aside from directly targeting viral and neoantigens, NK cells also function in conjunction with the humoral immune system by mediating a response known as antibody-dependent cell-mediated cytotoxicity (ADCC). ADCC occurs when target cells opsonized by antibodies are recognized by NK cells via the antibody Fc–Fc-gamma receptor (FcγR) interaction. Among the activation signals, activation through FcγRIIIa/CD16 initiates the most robust NK cell response and can even act as a mono-signal [6], which results in the release of cytotoxic granules and cytolysis of the target cells [2]. ADCC responses elicited by therapeutic vaccines or mediated by monoclonal antibodies (mAb) contribute to the protection against infection by influenza [3, 4] and Ebola [5, 7]. During SARS-CoV-2 infection, ADCC is detectable in patients as early as day 10 post-infection, and a higher ADCC response was found in those who have survived severe SARS-CoV-2 infection compared to those who succumbed to the disease [8]. Moreover, in animal models, anti-SARS-CoV-2 mAbs with limited neutralizing activity but potent ADCC mediated protection from infection [9]. Most importantly, for human immunodeficiency virus (HIV)-1 infection, ADCC plays a critical role in the control of infection in people living with HIV-1 [10–12], and during the RV144 efficacy studies, a strong ADCC response correlated with moderate efficacy in reducing the risk of infection by HIV-1[13, 14].

Human NK (HuNK) cells have been classically grouped into 3 subsets based on the expression of CD56 and CD16. CD56^dim^ CD16^pos^ NK cells are the predominant population, constituting around 90% of total NK cells in the peripheral blood [15]. This NK subset is characterized by high cytotoxicity, and they are the main mediator of the ADCC activity. Although conventionally regarded as a poor cytokine producer, recent studies indicate that CD56^dim^ subsets can function as a potent early producer of cytokines when engaged by target cells [16, 17]. On the other hand, CD56^bright^CD16^neg^ NK cells make up less than 10% of blood-derived NK cells and are known as “regulatory” NK cells with poor cytotoxic potential. They are mainly responsible for the production of cytokines such as IFNγ, TNF-α, GM-CSF, IL-10, and IL-13, depending on the precise conditions of stimulation [18, 19]. Lastly, the CD56^–^CD16^+^ NK cell subset is a rare population constituting 5.67% of the NK cell population in healthy peripheral blood, which is known to expand in patients who are chronically infected with Ebola, cytomegalovirus, HIV-1, and to a lesser extent, Herpes virus [20, 21]. Meta-analysis revealed that CD56^–^ cells could be further grouped into two subsets: a perforin^–^/CD94^–^/NKG2C^–^/NKp30^–^/CD57^–^ subset that showed very limited cytotoxicity against MHC-deficient target cells and another subset that expresses one or more of the perforin, CD57, NKG2C, NKp30, or CD94 markers and demonstrates cytotoxicity against MHC-deficient cells at a level similar to the CD56^dim^ cells [21].

During the last decades, an increasing number of studies have attempted to improve our understanding of NK cells by studying the overall transcriptomic profile of HuNK cells at a single-cell resolution [22–25]. This approach led to the identification of additional NK cell subpopulations, such as IFN-responding [23], transitional, active, and mature NK cells [22]. While these studies suggest that the heterogeneity of HuNK cells goes far beyond what has been previously characterized via surface receptor expression, they were conducted with cells at a steady state, which may not be representative of the transcriptomic profile of NK cells during a functional response such as ADCC. *In vitro* studies have shown that even in the presence of cell-activating reagents such as PMA and ionomycin, only up to 81% of cells undergo the degranulation of granzyme and perforin, suggesting that only a subset of NK cells is cytotoxic [26]. However, the majority of the studies published so far were performed on the bulk NK population without differentiating the responding cells from the rest of the non-responding cells. This lack of granularity limits our understanding of the specific NK cell subsets responsible for the cytotoxic and immunomodulatory functions during ADCC.

Non-human primates (NHP) are frequently used to assess the safety and efficacy of potential immunotherapeutic regimens, and studies have shown that like HuNK cells, NHP NK cells also play an important role in the control of microbial infections including tuberculosis [27], SARS-CoV-2 [28], and simian immunodeficiency virus (SIV) [29]. Rhesus macaques (RM) are the most frequently used NHP model in the field of HIV vaccine studies or preclinical trials, mainly due to their natural susceptibility to SIV and simian human immunodeficiency virus (SHIV), as well as their high resemblance to humans in terms of the pathophysiology of acquired immunodeficiency syndrome (AIDS) [1, 30]. Most importantly, ADCC-mediated protection against SIV/SHIV infection was also observed in the RM model [31–33], making rhesus macaque NK (RMNK) cells a valuable model for studying the effector population during ADCC response. Previous studies showed that RMNK cells can also be classified into 3 subsets: CD16^+^ RMNK cells that are functionally analogous to the cytotoxic CD56^dim^ CD16^+^ HuNK cells, CD56^+^ RMNK cells that resemble the CD56^bright^CD16^–^ HuNK cells, and lastly CD56^–^CD16^–^ double negative (DN) RMNK cells that seem to represent an intermediate population between CD56^+^ and CD16^+^ RMNK cells [1, 30].

Based on our review of previous literature, we determined that, in contrast to HuNK cells, the characterization of the RMNK cell transcriptomic profile is still limited. Therefore, to better interpret results from the preclinical studies involving the RM model, we sought to fill a critical gap in our understanding of the heterogeneity of RMNK cells and to identify features unique to the functional subsets that directly contribute to the antibody-mediated protections. To this end, we activated the RMNK cells through the Fc[.gamma]RIIIa/CD16-mediated mono-signal, sorted RMNK cells based on their functional involvement in the cytotoxic response, and then individually analyzed their transcriptomic profile. Our novel study showed that a specific subset of cytotoxic NK cells was responsible for high chemokine production and that the presence of antibodies alters the functional profile of NK cells even in the absence of measurable degranulation. Moreover, by isolating the Fc[.gamma]RIIIa/CD16 signaling from the co-stimulatory receptors and killer immunoglobulin-like receptors (KIR)-mediated signaling, we have established a crucial benchmark for evaluating anti-viral NK cell functions against infected cells, aiding in the understanding of the intricate signaling mechanisms originating from other activating receptors.

## METHODS

### Peripheral Blood Mononuclear Cells

Cryopreserved peripheral blood mononuclear cells (PBMCs) from 19 SIV-negative, cytomegalovirus-positive male Indian-origin RMs were analyzed in this study. The animals were housed, and the cells were harvested at the New Iberia Research Center. Animal experiments were approved by the New Iberia Primate Research Center IACUC (Protocol 2021-010-8823) and conducted in compliance with the principles described in the Guide for the Care and Use of Laboratory Animals [34]. Animals with B*17^+^ and B*08^+^ alleles were excluded from this cohort due to the protective effect of these alleles [35]. Due to the limitation of sample availability, only cells from male animals were analyzed in this study.

### ADCC-GranToxiLux Assay

The assay was performed as previously described [36–38]. Briefly, CEM.NKRCCR5 CD4^+^ T cell line (NIH AIDS Reagent Program, Division of AIDS, NIAID, NIH: from Dr. Alexandra Trkola) [39] coated with 5µg/mL of SHIV1157 QNE Y173H glycoprotein gp120 [32] were incubated with RM PBMC effector cells at an effector cell-to-target cell ratio of 30:1 with or without antibodies. We selected the SHIV 1157QNE Y173H envelope protein as our virus antigen target as this SHIV strain was previously used in a preclinical vaccine study [32]. The antibodies used were a combination of the C1C2 cluster A-region-specific antibodies JR4 [40] and DH677.3 [41], and V1V2- specific antibodies DH614.2 [42] and DH827 [43]. All antibodies were recombinantly produced as rhesus IgG1[42]. The combination of 4 rhesus mAbs was tested at a concentration of 1μg/ml each (4µg/mL of total IgG1). The influenza-specific rhesusized CH65-IgG1 (anti-HA) antibodies [44] were used at the equivalent concentration of 4µg/mL as negative controls. Samples were analyzed using flow cytometry and data were reported as the maximum proportion of cells positive for proteolytically active granzyme B (GzB) out of the total viable target cell population (maximum %GzB activity) after subtracting the background activity observed in wells containing effector and target cells in the absence of antibodies [37].

### Degranulation Assay

The assay was performed as previously described [45] with the following modifications. Cryopreserved RM PBMCs from 6 different animals were thawed and rested overnight before the assay in RPMI 1640 medium supplemented with antibiotics and 20% fetal bovine serum (R20). The effector cells and target cells were added to the wells with a ratio of 30:1 and incubated at 37°C and 5% CO for 6 hours in the presence of a cocktail of rhesusized ADCC-mediating mAbs listed in the ADCC-GranToxiLux assay section. The combination of 4 rhesus mAb was tested at a concentration of 1μg/mL each (4µg/mL of total IgG1). Protein transport inhibitors containing monensin (BD Bioscience, Cat#554724) and Brefeldin A (BD Bioscience, Cat#555029) were also added during the 6-hour incubation to maximize the signal detection. After the incubation, cells were then stained with surface markers listed in Supplementary Table 5 and resuspended in PBS for cell isolation by Fluorescence-activated cell sorting (FACS). All staining antibodies were tittered to maximize the signal-to-noise ratio as previously described [46].

### Flow Cytometry

Flow cytometry was used to assess the expression of surface receptors, chemokine, and percentage of CD107a^+^RM NK cells. Cryopreserved PBMC were incubated with anti-CD107a staining antibodies and protein transport inhibitors for 3 to 12 hours under one of the following 4 conditions: R10 only (Effector only), co-incubation with SHIV1157QNE Y173H gp120-coated CEM.NKR. CCR5 cell line(T+E), with SHIV SHIV1157QNE Y173H gp120-coated CEM.NKR.CCR5 cell line and ADCC mediating antibodies cocktail listed in ADCC-GranToxiLux assay section (ADCCAb), or with 20µg/mL of anti-CD16 antibodies (BD Biosciences, Cat# 555404) and 20µg/mL of goat anti-mouse secondary Fab (Jackson ImmnoResearch Laboratory, Cat# 115-006-003) (CD16CrossLink). Cells were then washed with PBS and stained with a viability marker (Fixable Aqua Dead Cell Stain Kit, ThermoFisher Scientific) before surface staining with fluorescently conjugated monoclonal antibodies (Supplementary Table 5) using standard techniques. Data were reported after performing background subtraction using readings from the effector-only condition.

### Luminex Assay

Custom-made Luminex bead array (ThermoFisher Scientific) specific for RM CCL3, CCL4, and CCL5 was used. RM PBMCs and gp120-coated NKR.CEM.CCR5 cell line were incubated with or without ADCC-mediating antibodies for 6 or 24 hours. RM PBMCs were also incubated with K562 cells for the same duration as the positive control. The culture supernatant was then diluted at 10-fold and added to the bead array kits according to the manufacturer's protocol to quantify the indicated analytes in duplicate sample wells. Assay plates were read using a FlexMAP 3D (Luminex) or BioPlex 200 instrument (BioRad) and data were analyzed using BioPlex Manager 6 (BioRad).

### scRNAseq Library Preparation

Cells were processed for scRNAseq using oil-in-droplet partitioning (10X Genomics). Briefly, cellular suspensions were loaded on a Chromium Single Cell Controller (10X Genomics) to generate single-cell beads in emulsion. Single-cell RNA-seq libraries were prepared using the Chromium Single Cell 3' v3 gel bead and library kit (10X Genomics). Sequencing-ready libraries were quantified with both a 2200 TapeStation using a D5000 screen tape (Aligent) and with Qubit using the dsDNA HS assay kit (ThermoFisher)

### Single-cell RNA Sequencing

Samples were sequenced on an Illumina NextSeq 500. Read lengths were 28 bp for read 1, 8 bp i7 index, and 91 bp read 2. Data generated with the 10X Chromium platform were processed using Single Cell Software Suite v5.0.1 (10X Genomics) using the Ensembl Mmul_10 v99 as the reference genome for aliments. The cell count ranged from 1,558 up to 10,089, with a minimum sequencing depth of 39,000 mean reads per cell (Supplementary Table 1). We down-sampled the cell count to 3,100 per RM (1550 CD107a^+^RMNK and 1550 CD107a^–^RMNK cells) and performed anchor-based integration [47, 48] to normalize the batch effect and ensure each animal contributes evenly to the clustering. This resulted in a total cell count of 18,600 cells encompassing all 6 animals.

### Transcriptomic Analysis

Graph-based cell clustering, dimensionality reduction, and data visualization were performed using the Seurat R package (version 5.0.1). For quality control, cells that exhibited >5% mitochondrial transcripts, expressed less than 500 unique transcripts, or expressed more than 2,500 unique transcripts were discarded. UMAP was performed with resolution = 0.6. This value was chosen because a further increase in the resolution did not result in additional functionally unique clusters. Differentially expressed transcripts were determined in the Seurat R package [47–49] utilizing the likelihood-ratio test for single-cell gene expression statistical tests with min.pct=0.1 and minimum log2 fold change= 0.25. Pathway analysis was performed using the *EnrichR* package using the top 100 most differentially expressed transcripts with log2 fold change >0.6. Graphics were generated using the Seurat and ggplot2 packages.

### Statistical Analysis

All statistical analyses were performed using R statistical software. Boxplots were used for data visualization and to summarize the distribution of various immune responses studied in this paper. The median is denoted by the midline of the box, and the 2 ends of the box denote 25^th^ and 75^th^ percentiles. The difference between the 2 percentiles is called the inter-quartile range (IQR). The whiskers denote the most extreme data points that are no more than 1.5 times IQR. Violin plots were used to show the spread and distribution of different levels of gene expression and module score. Mann–Whitney U test was used to compute the significance level of the GrantoxiLux assay, chemokine production, and chemokine concentration in culture supernatants. Two-tailed Student's *t*-test was used to compute the significance level of granzyme and perforin expression level, and one-tailed Student's *t*-test was used to compute the significance level of Module Score difference. Spearman's correlation coefficient was calculated for each cluster pair using the top 20 most differentially expressed transcripts. A significance level of 0.05 was used for all the statistical analyses.

### Data Availability

The datasets generated during and analyzed during the current study are available from the corresponding author upon reasonable request.

## RESULTS

### Isolation of ADCC-Mediating RMNK Cells for Single-cell RNA Sequencing

Cryopreserved PBMCs harvested from 6 experimentally naive adult male RMs were used for transcriptomic analysis (Supplementary Table 1). Flow cytometry analysis indicated that the average NK cell frequency was 2.54% of total RM PBMCs (1.32%-4.55%) (Supplementary Figure 1A, Supplementary Table 1). To gain insights into the transcriptomic profile of RMNK cells actively involved in ADCC, RM PBMCs were incubated with SHIV1157QNE Y173H gp120-coated CEM. NKR.CCR5 cells and a cocktail of human mAbs consisting of JR4 [40], DH677.3 [41], DH614.2 [42], and DH827 [43], all engineered to include the rhesus Fc region (rhesusized), at a previously optimized concentration [38] (see method section for details). ADCC-GranToxiLux [36, 37] assay showed the specific killing of target cells only in the presence of the anti-HIV-1-specific mAb cocktail but not the rhesusized flu-specific control antibodies CH65 (Supplementary Figure 1B, C), confirming that the mAb cocktail mediates HIV-1 envelope-specific ADCC response. To obtain RMNK cells that are directly involved in ADCC, we first gated on CD3^–^/CD14^–^/CD20^–^/CD8^+^/NKG2A/C^+^ RMNK cells as previously described [50, 51], then performed fluorescence-activated cell sorting (FACS) based on the expression of CD107a (Supplementary Figure 1A), which is a marker for degranulation [45, 52, 53]. Therefore, CD107a^+^ RMNK cells represent a degranulating population that has been recently involved in a cytotoxic response, while CD107a^–^RMNK cells represent cells that were not actively involved in the cytotoxic response [54].

### Unsupervised Clustering Identified 9 Subpopulations Within the ADCC Mediating RMNK Cells

After performing anchor-based integration [47, 48] on the total RMNK cells to normalize the batch effect and donor variation, dimensional reduction by UMAP and graph-based unsupervised clustering of the integrated dataset revealed a total of 13 initial clusters (Supplementary Figure 2A). To assess the expression of NK cell-associated transcripts in our dataset, we computed the module score for each of the clusters using the RhNK cell transcriptomic signature defined by Aid et al [55] consisting of 5,627 RMNK-associated transcripts. Out of the 13 initial clusters, 4 clusters (clusters 4, 5, 8, 11) showed lower RMNK cell module scores than the remaining clusters (Supplementary Figure 2B), as well as lower unique molecular identifier (UMI) count and total RNA read count (Supplementary Figure 2C) than the remaining clusters. Additionally, differential expression analysis also identified a cluster with high expression of T cell markers (CD3D/E/G, IL7R), indicative of T cell contaminations. Thus, for quality control purposes, these cells were removed from the downstream analysis, and the remaining cells were re-clustered for a better resolution.

**Figure 1.**
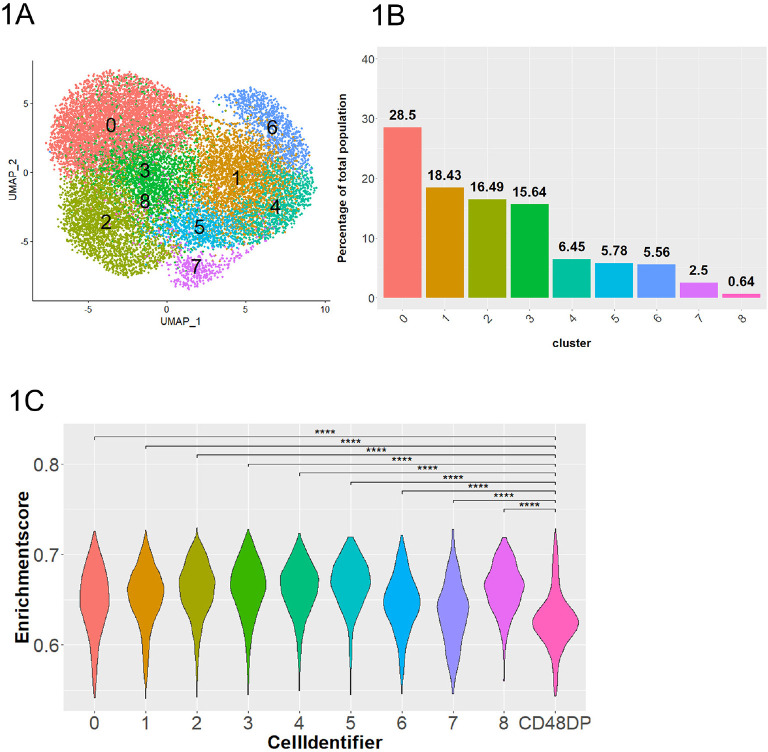
**Dimensional reduction and clustering of RMNK cells engaged by ADCC-mediated antibodies.** A) UMAP plot of RMNK cells colored and numbered by clusters after removal of low quality and non-NK cells. B) Proportion of each cluster relative to the total RMNK cells sequenced. C) NK module scores calculated using gene signatures identified by Aid et al and presented in violin plot. CD48DP is a CD4^+^ CD8^+^ RM T cell population used to compute the module score for non-NK population. Statistical significance was calculated with Student's 1-tailed *t*-test. ****P<0.0001.

This step resulted in a final cell count of 16,152 cells that were distributed among 9 clusters (Figure 1A, B). The 6 animals were evenly represented in every cluster, suggesting that the clustering is independent of batch-effect and donor-specific variation (Supplementary Figure 2D). As a control, we also obtained a RM T cell dataset [56] and applied the same module score analysis. All 9 clusters showed statistically higher module scores (*P*<0.0001) than that of the RM T cells (Figure 1C), confirming that our dataset consists of NK cells.

### Degranulating Cells Overall are Associated With an Activated Phenotype

**Figure 2.**
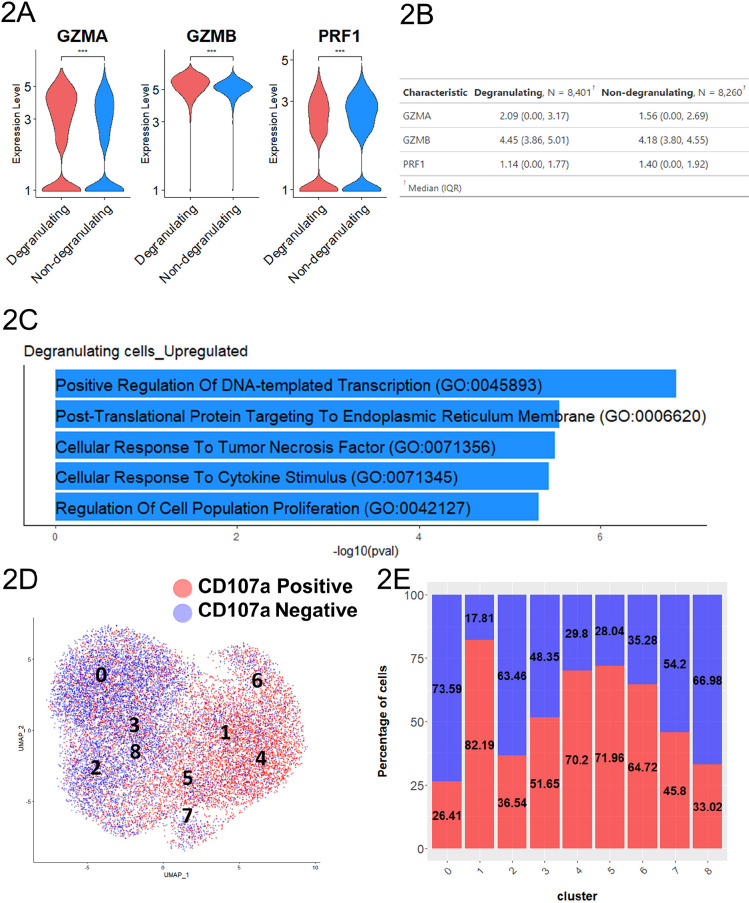
**Degranulating RMNK cells displayed a more activated phenotype.** A) Normalized expression level of transcripts associated with cytotoxicity. B) The summary statistics for the normalized expression of transcripts shown in 2A (N= number of the cell in each population. 2-tailed Student's *t*-test, ***P<0.001). C) Top 5 most enriched GO terms for degranulating cells compared to the non-degranulating cells. D) UMAP plot colored by degranulation status. E) Proportion of degranulating and non-degranulating cells within each cluster.

To gain insights into the functional difference between the degranulating CD107a^+^RMNK cells and the non-degranulating CD107a^–^RMNK cells, we first assessed the cytotoxic potential of the 2 populations by measuring the expression level of granzyme and perforin. As expected, the transcript level of granzyme genes (GZMA, GZMB) was higher in the CD107a^+^ population. However, perforin (PRF1), another key ADCC effector molecule, was expressed at a lower level in the CD107a^+^ cells (Figure 2A, B). To understand additional transcriptomic signatures unique to each of the populations, we performed differential expression analysis and selected transcripts that are expressed in at least 50% of either CD107a^+^ or CD107a^–^ population, with log2 fold change >1 or <-1 as the cut-off to focus our analysis on transcripts representative of the CD107a^+^ or CD107a^–^ population. This analysis identified 61 transcripts that were upregulated in CD107a^+^ cells relative to the CD107a^–^ cells, as well as 32 transcripts downregulated in CD107a^+^ cells (Supplementary Table 2). Transcripts upregulated in the CD107a^+^ population were associated with pathways including “Positive Regulation of DNA-templated Transcription” and “Negative Regulation of Programmed Cell Death” suggesting increased gene expression as well as cell survival (Figure 2C). Moreover, chemokine-related transcripts (XCL1, CCL3, CCL4L1) were also upregulated in the degranulating population compared to the non-degranulating counterparts, with XCL1 showing 8.7-fold (log_2_ Fc=3.12) difference in expression (Supplementary Table 2).

To assess the correlation between the killing due to ADCC activity and the expression of chemokine transcripts, we calculated the average expression levels of CCL3, CCL4L1, CCL5, and XCL1 by pseudo-bulking the degranulating NK cells by the animal ID. We then analyzed Spearman's correlation between the percentage of granzyme-positive target cells, which indicates the cell that would be killed, and the expression of chemokines. The comparison of these 2 readouts did not reveal significant correlations, possibly due to the difference in time of sample analyses for killing and collection for transcriptomic purposes (Supplementary Figure 3).

### Subsets of Degranulating Cells were Responsible for the Majority of the Chemokine Production

**Figure 3.**
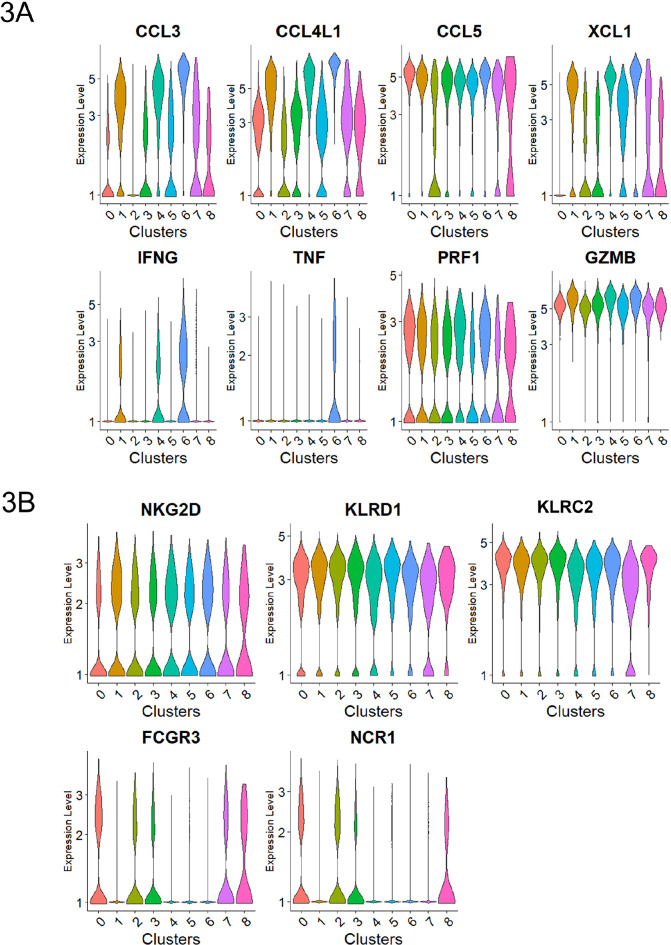
**Subset of degranulating cells displayed high chemokine production.** Normalized expression level of transcripts associated with A) cytokines and cytotoxicity and B) representative activating receptors.

While CD107a^+^RMNK cells as a whole could be characterized by higher chemokine productions and a more activated phenotype, considerable heterogeneity existed within these cells, as indicated by the presence of CD107a^+^RMNK across all 9 clusters (Figure 2D, E). Notably, CD107a^+^RMNK cells were predominant in clusters 1, 4, and 6, accounting for 82.19%, 70.2%, and 64.72% of the total cells in these respective clusters (Figure 2E). We observed that these clusters expressed high levels of chemokines and cytokine-associated transcripts as indicated by the enrichment of the regulation of cytokine production pathway (GO:0001817), driven primarily by the high expression of SRGN, RGCC, CRTAM, IRF8, and LITAF (Supplementary Figure 4). Among these clusters, cluster 6 stood out by exhibiting the highest levels of CCL3, CCL4L1, XCL1, and IFNγ expression, followed by clusters 1 and 4 (Figure 3A). This elevated chemokine production also contributed to the enrichment of the Cellular Response to Tumor Necrosis Factor pathway (GO:0071356) (Supplementary Figure 4).

It is noteworthy that the expression of TNFα itself was detected exclusively in Cluster 6 (Figure 3A).

A subset of CD107^+^RMNK cells was also present in clusters 0 and 2, which overlapped with the majority of CD107a^–^RMNK cells (Figure 2D, E). Except for *CCL5*, which showed a similar level of expression across all clusters, cells in these 2 clusters expressed a much lower level of chemokines and virtually no IFNγ (Figure 3A). These results suggest that after antibody engagement, only a subset of CD107a^+^RMNK cells respond with increased chemokine production.

Previous studies with HuNK cells have shown that the upregulation of co-stimulatory receptors such as NKG2C and NKG2D was one of the hallmarks of activated cytotoxic NK cells [57, 58]. While the expression levels of KLRC2(NKG2C), NKG2D, and KLRD1(CD94) transcripts in our RM dataset were similar across the 9 clusters, the level of FCGR3(CD16) and NCR1(Nkp46) in clusters 1, 4, and 6 were much lower compared to clusters 0 and 2 that were mostly dominated by CD107a^–^RMNK cells (Figure 3B). Clusters 1, 4, 6 primarily consist of CD107a^+^ cells, which respond to antibody-dependent activation via the CD16 receptor. Given that ADCC antibodies are the sole activation source, these CD107a^+^ cells likely have expressed CD16 before activation and degranulation. HuNK cells are known to downregulate CD16 and NKp46 after activation [59, 60]. Our data indicate that the receptor downregulation is also true at the transcript level, and this regulation is conserved between humans and rhesus.

### An Optimal Level of Antibody Engagement is Required for Potent Chemokine Production *In Vitro*.

**Figure 4.**
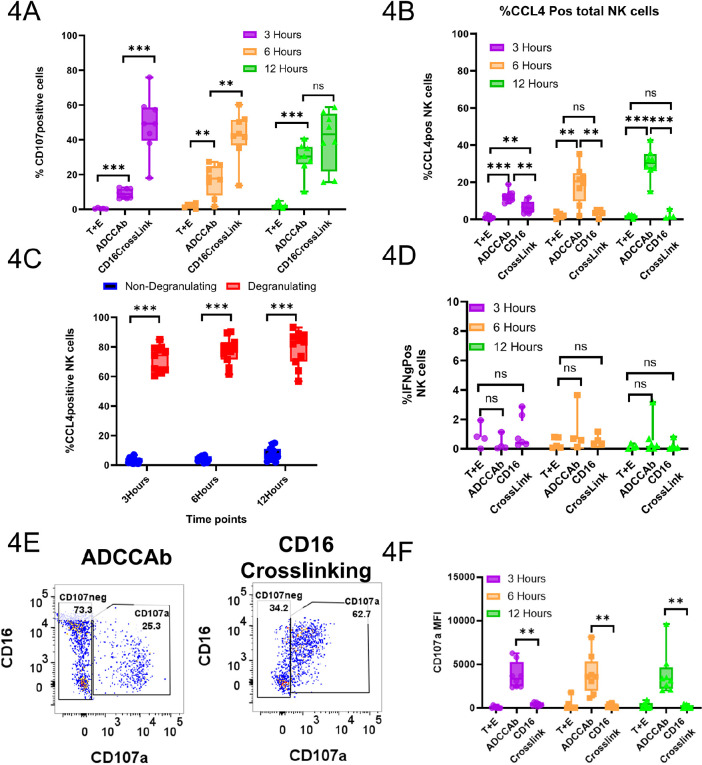
**Flow cytometry confirms the antibody-mediated chemokine production by RhNK cells at protein level.** A) Percentage of NK cells that were positive for the CCL4 after intracellular staining. T+E=Target and PBMCs only; ADCCAb=Target cells and PBMCs incubated in the presence of ADCC-mediating antibodies; CD16 Crosslink= Opsonization of RhNK cells by mouse anti-human CD16 antibodies followed by crosslinking with secondary goat anti-mouse F(ab)'2. B) Percentage of CCL4^+^ cells within the degranulating and non-degranlating NK cell subsets from the ADCCAb group. C) Percentage of cells positive for IFNγ after intracellular staining. D) Percentage of cells positive for CD107a molecules. E) Representative dot plot for the expression of CD107a. F) Median fluorescence intensity of CD107a. All data were reported after background subtraction using PBC-only conditions. Mann-Whitney U test, n.s.=not significant; *P<0.05; ** *P* <0.01; ***P <0.001.

To verify if the upregulation of chemokine production by subsets of CD107a^+^RMNK cells is also true at the protein level, we obtained PBMCs from 8 other RMs, performed intracellular staining of CCL4 and IFNγ, and analyzed their expression at 3-, 6-, and 12-hour time points using flow cytometry. RMNK cells were co-incubated with target cells with or without ADCC-mediating antibodies (ADCCAbs) or with anti-CD16 antibodies. CCL4 production began as early as 3 hours in the presence of ADCCAbs and steadily increased over the next 9 hours, while CCL4 production was negligible when the ADCCAbs were absent (Figure 4A). Furthermore, in the presence of the ADCCAbs, the majority of the degranulating cells produced CCL4 at all 3 time points, while CCL4 production by non-degranulating cells was minimal (Figure 4B). IFNγ production, on the other hand, was very low across all the experimental conditions and time points (Figure 4C).

While the addition of anti-CD16 antibodies yielded a significantly higher percentage of CD107a^+^ cells than the addition of ADCCAbs across all 3 time points (Figure 4D, E), the average CD107a median fluorescence intensity (MFI) of CD107a^+^RMNK cells from the CD16 crosslinking group was significantly lower than that of cells from the ADCCAbs (3 hours: 337 vs 3885; 6 hours: 185 vs 3611; 12 hours: 124 vs 3278) (Figure 4E, F), and the CCL4 production signature was much weaker as well (Figure 4A). These data suggest that CD107a^+^RMNK cells are indeed responsible for the majority of chemokine production within the NK cell population and that the quality and quantity of receptor engagement are important determinants of the cellular response.

Although intracellular staining showed that antibody engagement resulted in increased chemokine production, chemokines are soluble factors that need to be released extracellularly to function. To quantify the amount of chemokine that is accessible to other cells, we performed Luminex assays with beads that are specific to rhesus CCL3, CCL4, and CCL5 (ThermoFisher Scientific) and measured the chemokine concentration in the PBMC culture supernatant at the 6-hour and 24-hour timepoints. We excluded IFNγ because its concentration was below the limit of detection during our pilot runs. While the median chemokine concentration of CCL3 and CCL4 in the culture supernatant was higher at both time points in the presence of the antibody compared to the target and effector cell-only condition, the difference was not statistically significant (Supplementary Figure 5, Supplementary Table 4). For CCL5, the addition of antibodies resulted in a lower median chemokine concentration, although the difference was also not statistically significant.

## FEATURES ASSOCIATED WITH THE REMAINING CLUSTERS

Cluster 0, cluster 2, and the 3 chemokine producer clusters (1, 4, 6) accounted for 73% of the total CD107a^+^RMNK cells, and the remaining 27% of CD107a^+^RMNK cells were unevenly distributed among clusters 3, 5, 7, and 8 (Figure 1A, 2B). Cluster 3 was one of the clusters consisting of an equal proportion of CD107a^+^ and CD107a^–^RMNK cells, accounting for 8.0% of the total CD107a^+^RMNK cells. Based on the transcriptomic profile, cluster 3 is closely related to cluster 0 as indicated by a high correlation value (Spearman's correlation, 0.954. Supplementary Figure 6). However, cells in cluster 3 expressed *XCL1* that was absent in cluster 0 (Figure 3A).

**Figure 5.**
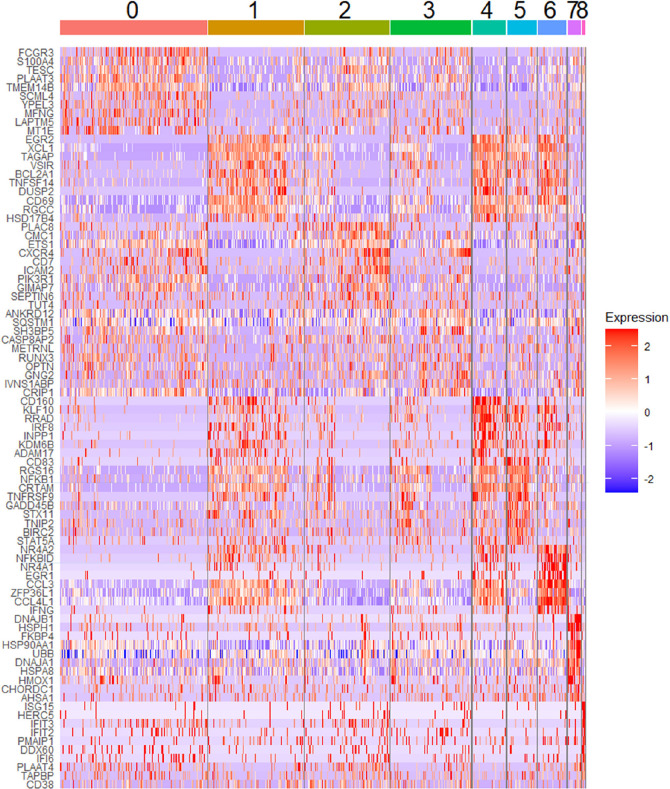
**Heat map showing top 10 most upregulated transcripts for total RMNK cells in each cluster**.

Cluster 5 is a smaller cluster accounting for 4.2% of the total CD107a^+^RMNK cells. Compared to the chemokine-producing clusters (1, 4, 6), cluster 5 exhibits a lower expression of CCL3 and CCL4L1, but its XCL1 expression was comparable to that of cluster 1 and much higher than that of clusters 0, 2, and 3 (Figure 3A). Another feature that distinguished cluster 5 was the upregulation of I-κB kinase/NF-κB signaling pathway (GO:0007249) (Supplementary Figure 4) driven by the highest NF-kB subunit 1 (NFKB1) expression as well as the lowest level of NFKBID expression among all the clusters (Figure 5). NF-κB is essential for the formation of mature NK cells [61], while NFKBID plays an inhibitory role in the NF-κB pathways inside mature cells as part of the negative feedback loop. The expression pattern of these 2 transcripts in cluster 5 suggests that the cluster corresponds to a population of cells that are currently in the process of transitioning into mature NK cells but have not achieved the terminal stage. Of note, cluster 6, which was the most potent producer of chemokines and IFNγ, showed the highest level of NKBID, followed by clusters 1 and 4, suggesting that these 3 clusters consist of cells with subtle differences in maturity and/or functional stages. While the specific signaling cascade of NFKBID in NK cells has not been elucidated yet, studies have shown that the engagement of TCR and TLR results in the up-regulation of NFKBID in mature CD4^+^ T cells and mature macrophages, respectively [62, 63]. The elevated NFKBID expression in clusters 1, 4, and 6 may similarly result from CD16 engagement.

Cluster 7 accounted for 1.6% of the total CD107a^+^RMNK cell population and was the only group of cells that showed high expression of heat shock proteins (HSP90AA1, HSPH1, HSP8A, DNAJA1, DNAJB1) (Figure 5), which participate in protein folding and refolding and are known to be upregulated after oxidative damage and heat stress [64]. External stress due to the freeze-thaw cycle, cell preparation, and FACS may have contributed to the emergence of this population. Of note, cells in cluster 7 expressed surface receptors (NKG2C, NKG2D) and lytic molecules (GZMB. PRF1) at a level comparable to the remaining clusters, suggesting that they are still functional and possibly in the process of recovering from the stresses.

Cluster 8 was the smallest cluster, accounting for only 0.21% of total CD107a^+^RMNK cells. These cells exclusively expressed several interferon-stimulated genes (ISGs) including IFIT2, IFT3, and ISG15 (Figure 5), suggesting IFN-mediated activation of RMNK cells. This observation was confirmed with pathway enrichment analysis, where “defense response to virus,” “negative regulation of viral replication and processes,” and “response to interferon beta” were among the top 5 most upregulated pathways in cluster 8 (Supplementary Table 3). This population has also been reported by Yang et al in HuNK cells [22]. In that study, this cell subset mostly consisted of cells from a single male donor. In our data, cluster 8 consisted of cells from all 6 animals, suggesting that this cluster is a distinct RMNK cell subpopulation and not a donor-specific feature.

### The Presence of Antibodies Results in the Activation of Cells in the Absence of Degranulation

**Figure 6.**
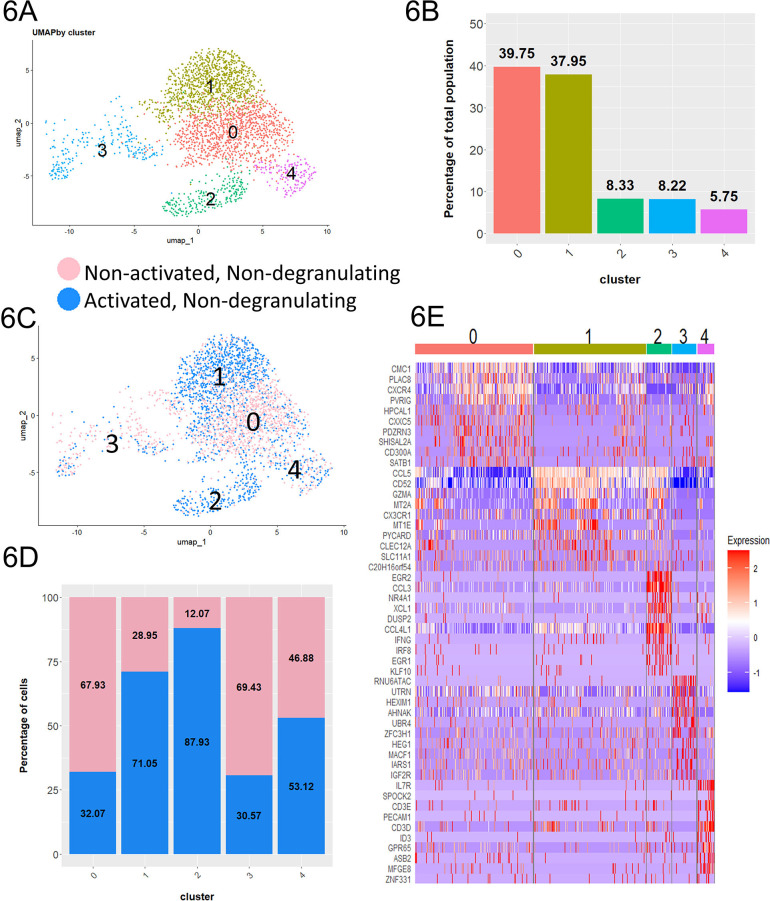
**ADCC-mediating antibodies contribute to the activation of cells without inducing degranulation.** A) UMAP plot consisting of non-activated non-degranulating cells incubated without antibodies and activated non-degranulating cells incubated with ADCC-mediating antibodies. B) Proportion of each cluster relative to the total integrated population. C) UMAP plot colored by condition. D) Proportion of the non-degranulating and non-activated cells within each cluster. E) Heat map showing top 10 most upregulated transcripts in each cluster.

While non-degranulating cells were not directly involved in the cytotoxic response, they were still in full contact with ADCC-mediating antibodies during the co-incubation and could be potentially activated to mediate other functions. To investigate the potential effect of antibodies on the non-degranulating CD107a^–^RMNK cells, we compared the CD107a^–^RMNK cells to the NK cells that had been incubated with target cells in the absence of ADCC mediating antibodies. Due to the lack of ADCC-mediating antibodies, these cells are minimally activated in terms of both cytotoxicity and chemokine production (Supplementary Figure 1B, Figure 4A, D, F) and for simplicity are denoted as “non-activated non-degranulating cells,” while the CD107a^–^RMNK cells incubated in the presence of ADCC-mediating Abs were labeled as “activated non-degranulating cells.” The integration of the above two populations yielded 2,785 cells that formed 5 clusters (Figure 6A, B). Among those, cluster 1 and cluster 2 were highly enriched with activated non-degranulating cells, suggesting that these 2 groups of cells resulted from the presence of antibodies (Figure 6C, D). Cluster 1 accounted for 37.95% of the population. While CCL5 was the only chemokine that was upregulated in cluster 1, it showed a high expression of markers of cytotoxicity (GZMA) as well as markers associated with maturation and activation (CX3CR1, S100A4, CD52) [23, 65, 66], representing a functionally activated cell population.

On the other hand, cluster 2 represents 8.33% of the total population (Figure 6B) and showed up-regulation of CCL3, CCL4L1, CCL5, and XCL1, and was the only cluster expressing IFNγ (Figure 6E, Supplementary Figure 7A), representing a rare, non-degranulating chemokine producer. Interestingly, the overall transcript level of FCGR3(CD16) was lower in these chemokine-producing cells compared to the cells in clusters 0 and 1. To assess whether the expression of IFNγ was triggered by CD16-mediated signaling, we investigated the FCGR3 expression profiles of the cells in cluster 2 that were positive for IFNγ. The majority of these cells were not expressing FCGR3 transcripts (Figure 7B). However, 88% of the cells in cluster 2 belonged to the activated non-degranulating cell group, which suggests that the presence of antibodies played a major role in the generation of this population. The ADCC-mediating antibodies may have engaged the CD16 receptor directly, which subsequently resulted in the downregulation of CD16 expression, or the soluble factors produced by surrounding activated degranulating cells might have contributed to the expression of IFNγ in cluster 2. Altogether, the enrichment of functionally active cells in the activated non-degranulating population as compared to its non-activated counterparts suggests that ADCC-mediating antibodies can activate NK cells even in the absence of measurable degranulation.

## DISCUSSION

ADCC responses have been implicated to play a crucial role in the control of a wide range of viral infections not limited to HIV-1 but also Ebola, influenza, and COVID-19 [3–5, 7, 8, 28]. RMs have been an excellent model for candidate AIDS vaccine regimens [67–69], including those demonstrating the critical role played by ADCC in controlling viral replication or infection [32,33]. However, our knowledge of the RMNK cells during ADCC responses is currently very limited, and to the best of our knowledge, none of those studies has performed analysis at single-cell resolution on the RMNK cell subsets actively involved in ADCC response. To fill this gap, we engaged the RMNK cells with antibody-coated target cells, which activate the NK cells via Fcγ-RIIIa/CD16 with minimal costimulatory receptors and KIR/KIR-ligand mediated signaling. After this stimulation, we sorted and performed single-cell RNA sequencing on RMNK cells collected from 6 different animals. We then performed unbiased clustering to identify the heterogeneity of RMNK cells that have been engaged by antibodies to recognize the target cells in an antigen-specific manner. Our analysis revealed that in addition to inducing ADCC, antibody engagement also resulted in the potent production of both CC chemokines CCL3 and CCL4L1 as well as C chemokine XCL1 by degranulating cells, which was further confirmed via flow cytometry assays. Of note, sequencing data indicates that only subsets of CD107a^+^RMNK cells were capable of producing chemokines at a high level, except for the CC chemokine CCL5, whose transcript was expressed at a similar level across all clusters.

During the cell preparation for sequencing and intracellular staining of chemokines, protein transport inhibitors (PTIs) containing brefeldin and monensin were added to the culture to enhance the signal intensity of labeled CD107a and chemokines. Monensin reduces the degradation of fluorescently labeled CD107a by neutralizing the pH of endosomal and lysosomal compartments, while brefeldin blocks the intracellular protein transport processes to promote the accumulation of cytokines inside the cells. Additionally, the degranulating cells and non-degranulating cells analyzed in this study were acquired from the same PBMC cultures and received the same amount of PTIs during incubations. Since both reagents function post-transcriptionally and both cell populations used to identify transcriptomic profiles were exposed to the PTI, we do not have evidence that the addition of protein transport inhibitors altered the transcriptomic profile of the RM NK cells.

In the current study, we did not observe a significant correlation between the potency of ADCC activity and the expression of transcripts associated with chemokines (CCL3, CCL4L1, CCL5, XCL1). This is likely because the GranToxiLux assay only measures the immediate early response that takes place within 2 hours of incubation with target cells, while chemokine production occurs over a longer span, and the expression was measured at the 6-hour time point.

Although artificial crosslinking of the CD16 receptor resulted in a significantly higher number of cells to degranulate, the CD107a MFI was much lower than that of the ADCCAb condition. CD107a is expressed on the inner membrane of cytotoxic granules and becomes accessible to the stain only when the granules fuse with the cell membrane after exocytosis [52, 70]. Thus, lower MFI suggests that although a higher proportion of the NK cells are degranulating, the amount of cytotoxic granules released by each of those degranulating cells is lower than those induced by ADCCAb. Both ADCC-mediating mAb cocktail and anti-CD16 crosslinking antibodies activate NK cells by engaging the Fc[.gamma]RIIIa/CD16 receptor. However, they interact with the cells in vastly different ways. ADCC response requires the formation of immune complexes that involve cell-to-cell contact [71, 72]. In this study, effector cells were in excess compared to the target cells (E: T ratio 30:1), such that the NK cells only encounter a few target cells at any given time and the signaling takes place in a limited, polarized manner [73]. On the other hand, anti-CD16 and the secondary goat anti-mouse F(ab)'2 antibodies were added to the media in excess (20µg/mL) as compared to the mAb cocktail (4µg/mL), which allowed the anti-CD16 antibody to freely bind NK cells from any direction. This may result in more frequent receptor engagement in a non-polarized manner. Next, the CEM.NKR.CCR5 cell line is known to express HLA-E and CD48 [74], which serve as the ligand for activating receptors NKG2C and CD2 respectively. Additionally, stress-induced molecules on the target cells may also engage the NKG2D receptor. Cell-to-cell contact may provide additional signals through these co-stimulatory receptors, but such signaling is absent during activation through anti-CD16 antibodies and secondary anti-mouse F(ab)'2. Altogether, our results suggest that NK cell responses vary based on the frequency and quality of the receptor engagement, and in some cases, the over-activation of NK cells results in a skewed function.

Re-clustering of activated non-degranulating RMNK cells with non-activated non-degranulating RMNK cells showed that in the presence of ADCC-mediating antibodies, a small subset of non-degranulating RMNK cells is also capable of chemokine production. The production of chemokines could be either triggered by direct engagement of CD16 receptors by antibodies or by cytokines produced by neighboring cells. As shown in Figure 3C, IFNγ production by activated NK cells is minimal within 12 hours, and IFNγ produced by non-NK CD3^+^ cells was even lower (Supplementary Figure 8). While we cannot exclude the contribution of other soluble factors that were not monitored in the assay, we consider IFNγ as the proxy for the production of other cytokines by non-NK cells. Thus, it is more likely that CD16-mediated signaling is the major driver of chemokine production within these non-degranulating cells.

In this study, the transcriptomic profiles were assessed at the 6-hour time point. However, the flow cytometry data suggest that RMNK cell degranulation steadily increased at least until the 12-hour time point (Figure 4D). Thus, while the non-degranulating, chemokine-producing cells may represent a rare cell subset with unique properties, it is also possible that they correspond to an immature non-cytotoxic population that acquires cytotoxic properties and degranulates at later time points. If the latter case is true, this would suggest that chemokine production could be an early response that either precedes the acquisition of or is independent of cytotoxic function. Due to a lack of appropriate reagents that effectively bind to rhesus CCL3 and XCL1 molecules, the expression of these 2 markers could not be directly assessed with flow cytometry. However, based on published literature [17, 75] as well as the similarity in the expression pattern of these transcripts with that of *CCL4L1* in our study, the production of CCL3 and XCL1 molecules should follow the same trend at the protein level.

Chemokines are soluble factors responsible for the recruitment of a wide range of immune cells via chemotaxis. XCL1 recruits conventional dendritic cells, while CC chemokines are responsible for the recruitment of lymphocytes, monocytes, eosinophils, and basophils [76]. In addition to this immunomodulatory role, CCL3, CCL4, and CCL5 belong to the beta chemokine family that uses CCR5 as the receptor [76]. Because of this property, several studies have shown that these chemokines play an important role in inhibiting HIV-1 entry and replication [75, 77–79]. Previous studies involving recombinant human beta chemokines showed that a concentration of 50ng/mL [78] was sufficient to significantly inhibit multiple strains of HIV-1 replication in human PBMC *in vitro*. Due to the lack of reagents suitable for the isolation of RMNK cells, we measured the chemokine concentration in the culture supernatant of RM PBMC in the presence of target cells and ADCC-mediating antibodies. The median concentration was between 57.65pg/mL and 894.47pg/mL across the 2 time points (Supplementary Table 4). These concentrations are lower than those previously reported using purified HuNK cells [75]. However, this discrepancy is likely due to the shorter incubation time and consistent utilization of chemokines by surrounding non-NK cells, leading to an underestimation of the amount produced in our experimental conditions. At present, it is still unknown if the number of chemokines produced by antibody-engaged RMNK cells is sufficient to achieve the inhibitory effect, and we plan to design studies that can evaluate viral outgrowth using RMNK cells with chemokine knockout and carefully designed conditions for the infection of cellular targets.

Aside from the chemokine-producing population, we also identified several clusters with distinctive features that have also been reported in human studies [22, 23], such as stress-induced RMNK cells and IFN-activated RMNK cells, suggesting that in the presence of ADCC-mediating antibodies, RMNK cells exhibit similar degrees of heterogeneity as that of humans. A limitation of our study is related to our inability to assign clusters to the well-established NK cell subsets based on the CD56 and CD16 expression. This is largely due to the CD16-mediated activation, which results in the CD16 downregulation [59]. Additionally, previous studies have reported that unlike HuNK cells, in which CD56 expression was variable but sufficiently high to be detected, CD56 expression in RMNK cells is very low [30] and was mostly below the threshold of detection. To our knowledge, no human studies have been performed in the context of degranulation status, so future work with HuNK cells activated with ADCC-mediating mAbs needs to be performed to be able to directly compare human and RMNK cells.

In summary, we characterized, at single-cell resolution, the functional subset of RMNK cells that were directly involved in the ADCC response. Our results highlight the heterogeneity within the RMNK cells after engagement by ADCC-mediating antibodies, demonstrating that a subset of engaged cells can contribute to a broader anti-viral function through the production of chemokines, which can either directly inhibit virus replication or redirect other immune cell subsets to the site of infection. This work can provide valuable insights into the complex heterogeneity of RMNK cells, which could be utilized to interrogate the species-specific protective responses in vaccines and immunoprophylaxis studies and advance our understanding of the similarities and differences between humans and rhesus macaques. By triggering ADCC through target cells opsonized with antibodies, we have isolated the Fc[.gamma]RIIIa/CD16 signaling from the co-stimulatory receptors and KIR-mediated signaling. These data could serve as an important benchmark for anti-viral NK cell functions against infected cells to understand the complexity of the signals originating from other activating receptors.

## SUPPLEMENTARY DATA

Supplementary materials are available at the *Pathogens and Immunity* website. Supplementary data may be provided by the authors to benefit the reader. Supplementary data are not copyedited and are the sole responsibility of the authors. Questions or comments related to supplementary materials should be addressed to the corresponding author.
