# Supplementary material for: Increased Chemokine Production is a Hallmark of Rhesus Macaque Natural Killer Cells Mediating Robust Anti-HIV Envelope-Specific Antibody-Dependent Cell-Mediated Cytotoxicity
Source: Pathog Immun. 2025 Jan 23;10(1):49–79. doi: 10.20411/pai.v10i1.734 (PMC11792536; doi:10.20411/pai.v10i1.734)
Supplement: Supplementary Figures [file pai-10-049-s01.pdf]

PATHOGENS AND IMMUNITY
In Association with Case Western Reserve University and The Richard J. Fasenmyer Foundation

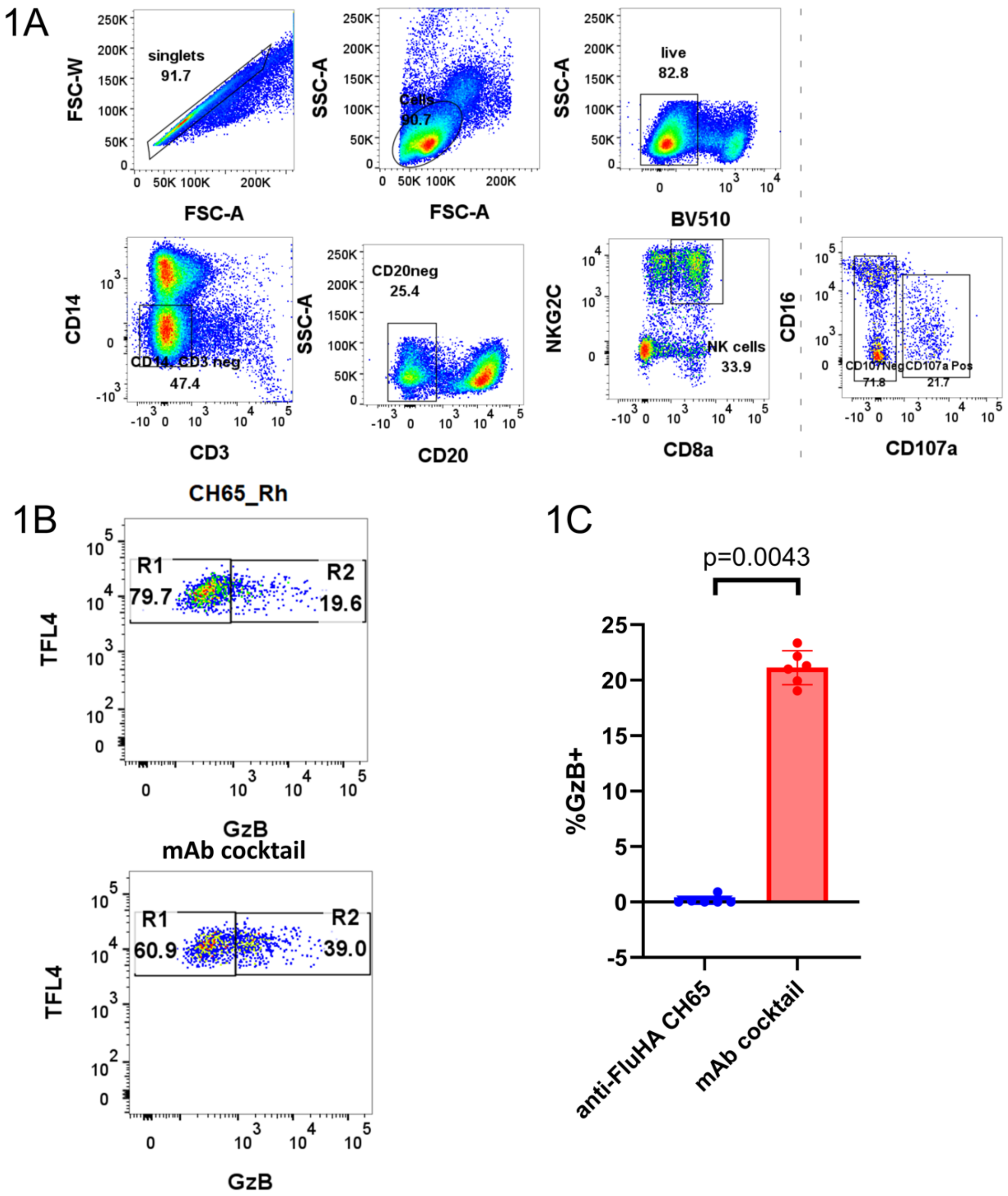

Supplementary Figure 1. Single RNA sequencing was performed on RM NK cells. A) Gating strategy used for the sorting of RhNK cells. B) Representative dot plots from the ADCC-GranToxiLux assay. C) ADCC activity quantified using ADCC-GranToxiLux assay. D) Y-axis represent Maximum proportion of target cells positive for proteolytically active granzyme B (GzB) after background subtraction. n=6, statistical significance was calculated using Mann–Whitney U test.

Nohara J, Evangelous T, Berry M, Beck W, Mudrak S, Jha S, Reeves RK, Wiehe KJ, Pollara J, Tomaras GD, Bradley T, Ferrari G. Increased Chemokine Production is a Hallmark of Rhesus Macaque Natural Killer Cells Mediating Robust Anti-HIV Envelope Specific Antibody-Dependent Cell-Mediated Cytotoxicity. *Pathogens and Immunity*. 2024;10(1):49–79. doi: 10.20411/pai.v10i1.734

PATHOGENS AND IMMUNITY
IN ASSOCIATION WITH CASE WESTERN RESERVE UNIVERSITY AND THE RICHARD J. FASENMYER FOUNDATION

2A

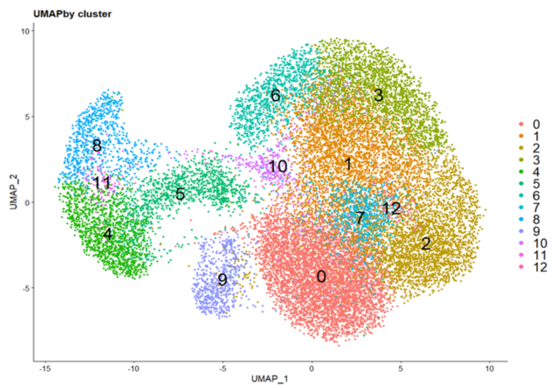

2B

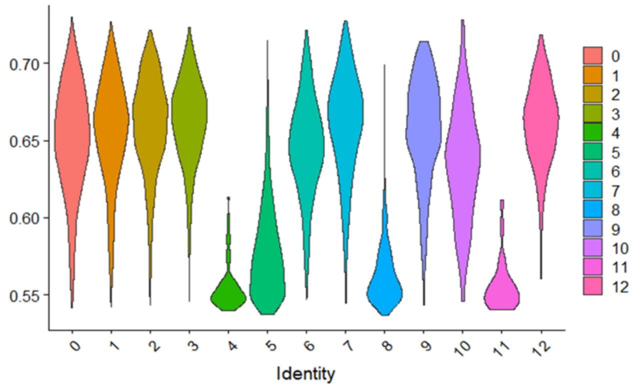

2C

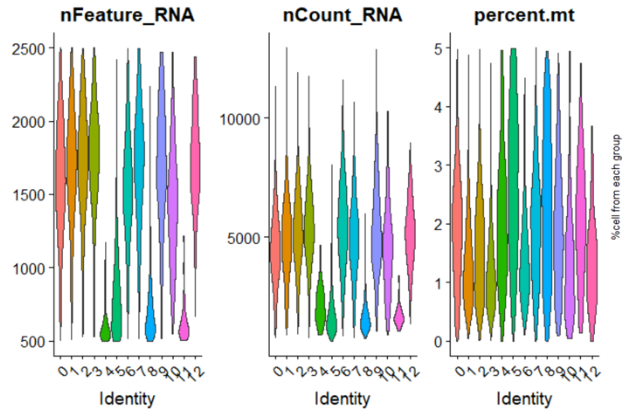

2D

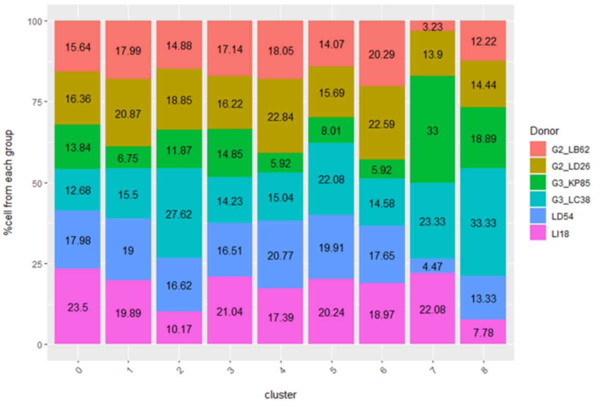

Supplementary Figure 2. Data before the removal of low-quality cells. A) UMAP plot displaying the clustering in the presence of low-quality cells. B) NK module score calculated using gene signatures identified by Aid et al. C) Violin plot showing the UMI count, total RNA read, and percentage of Mitochondrial transcripts relative to the total transcript count. D) Distribution of animals within each cluster. Each color represents a different animal.

Nohara J, Evangelous T, Berry M, Beck W, Mudrak S, Jha S, Reeves RK, Wiehe KJ, Pollara J, Tomaras GD, Bradley T, Ferrari G. Increased Chemokine Production is a Hallmark of Rhesus Macaque Natural Killer Cells Mediating Robust Anti-HIV Envelope Specific Antibody-Dependent Cell-Mediated Cytotoxicity. *Pathogens and Immunity*. 2024;10(1):49–79. doi: 10.20411/pai.v10i1.734

PATHOGENS AND IMMUNITY
IN ASSOCIATION WITH CASE WESTERN RESERVE UNIVERSITY AND THE RICHARD J. FASENMYER FOUNDATION

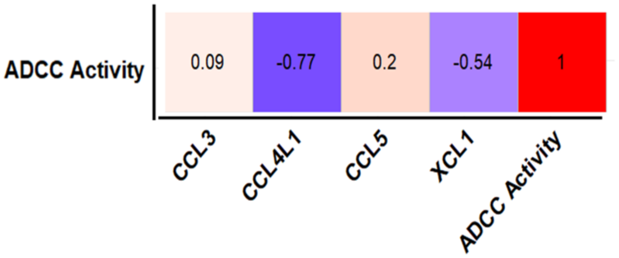

Supplementary Figure 3. Spearman's Correlation between ADCC activity and Chemokine expression.

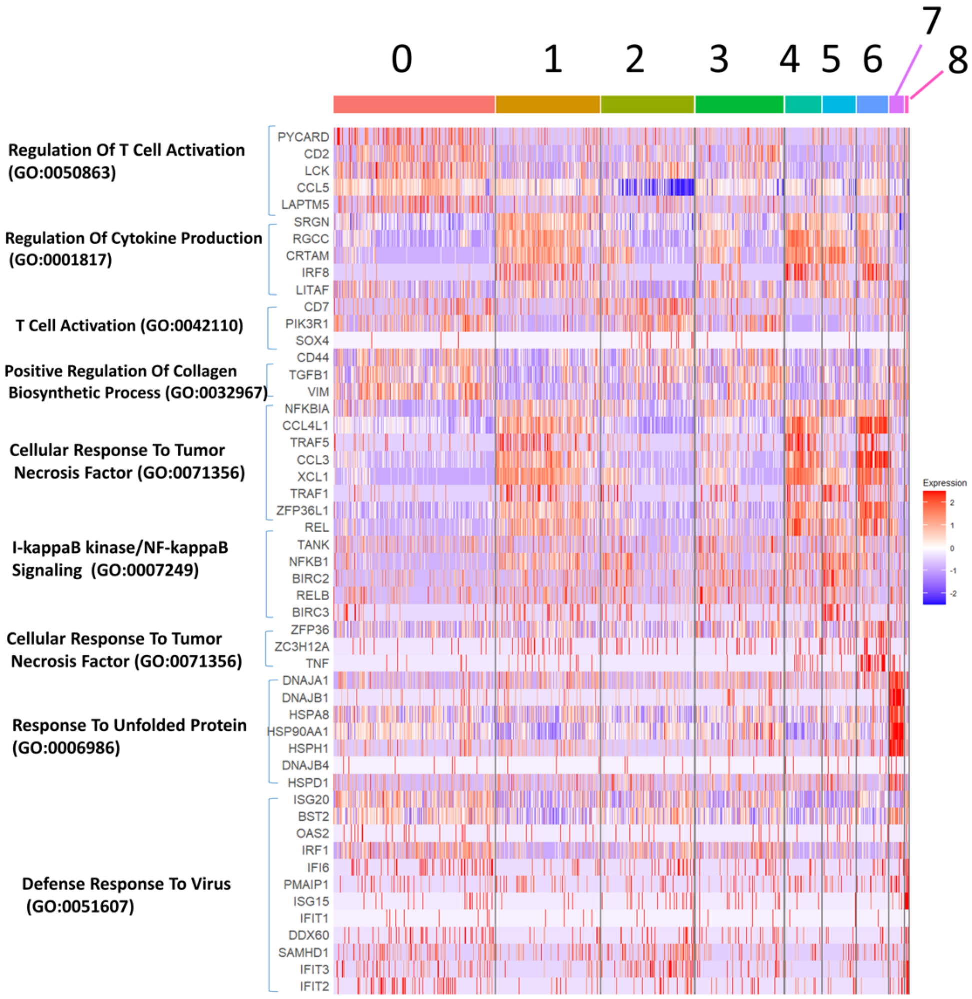

Supplementary Figure 4. Heatmap showing the leading-edge genes for the most enriched pathway of 9 clusters consisting of total RMNK cells. Leading-edge genes appearing in more than one pathway are only listed once under the pathway they first appeared.

Nohara J, Evangelous T, Berry M, Beck W, Mudrak S, Jha S, Reeves RK, Wiehe KJ, Pollara J, Tomaras GD, Bradley T, Ferrari G. Increased Chemokine Production is a Hallmark of Rhesus Macaque Natural Killer Cells Mediating Robust Anti-HIV Envelope Specific Antibody-Dependent Cell-Mediated Cytotoxicity. *Pathogens and Immunity*. 2024;10(1):49–79. doi: 10.20411/pai.v10i1.734

PATHOGENS AND IMMUNITY
IN ASSOCIATION WITH CASE WESTERN RESERVE UNIVERSITY AND THE RICHARD J. FASENMYER FOUNDATION

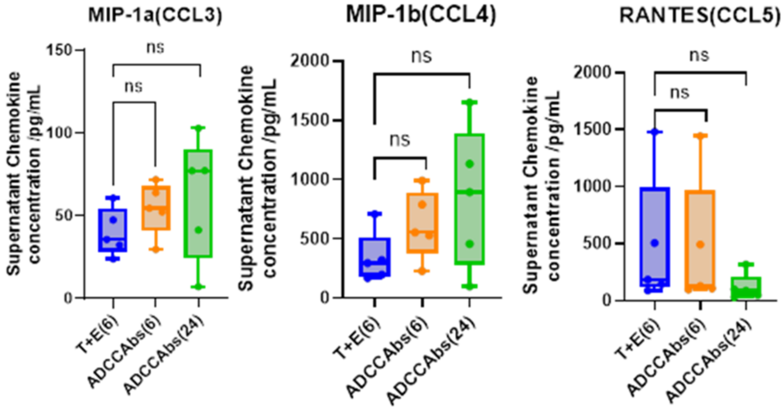

Supplementary Figure 5. Chemokine concentration in the RM PBMC culture supernatant. Cells were incubated for 6 or 24 hours with or without antibodies and Luminex assay was performed using custom-designed beads specific for Rhesus chemokines. N=5, Mann–Whitney U test. ns= not significant.

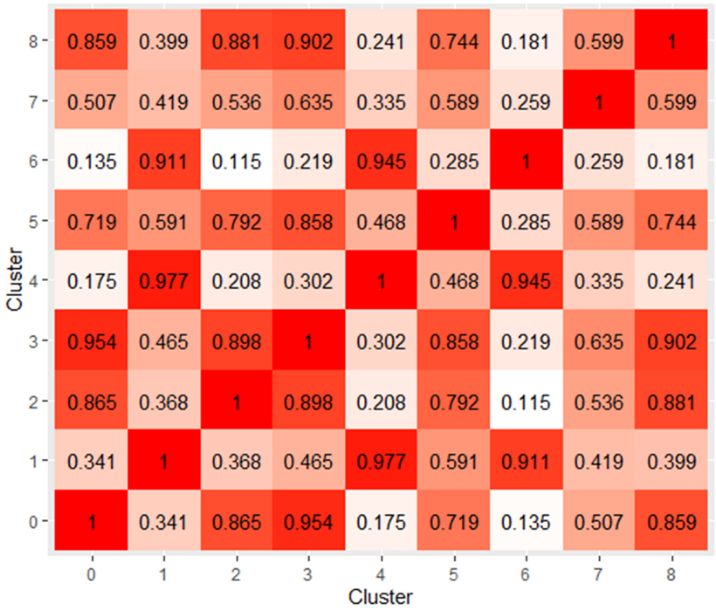

Supplementary Figure 6. Spearman's correlation using the top 20 most differentially expressed transcripts in each cluster.

Nohara J, Evangelous T, Berry M, Beck W, Mudrak S, Jha S, Reeves RK, Wiehe KJ, Pollara J, Tomaras GD, Bradley T, Ferrari G. Increased Chemokine Production is a Hallmark of Rhesus Macaque Natural Killer Cells Mediating Robust Anti-HIV Envelope Specific Antibody-Dependent Cell-Mediated Cytotoxicity. *Pathogens and Immunity*. 2024;10(1):49–79. doi: 10.20411/pai.v10i1.734

PATHOGENS AND IMMUNITY
IN ASSOCIATION WITH CASE WESTERN RESERVE UNIVERSITY AND THE RICHARD J. FASENMYER FOUNDATION

7A

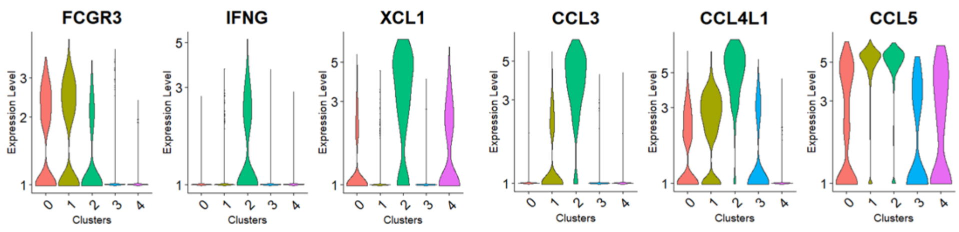

7B

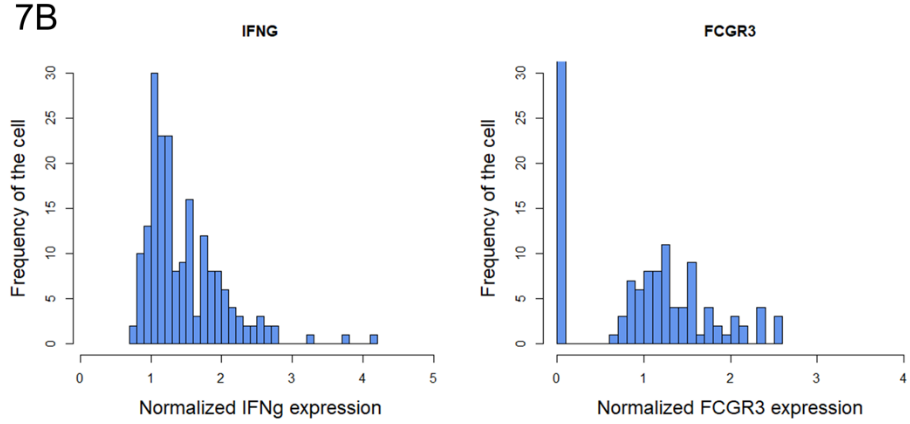

Supplementary Figure 7. Expression data for non-activated non-degranulating cells and activated non-degranulating cells. A) Normalized expression of CD16 and chemokine transcripts. B) Distribution of the IFNγ and FCGR3 expression in the IFNγ+ cells in cluster 2. Log normalization was performed on the raw transcripts count with a scale factor of 10000. The y-axis for FCGR3 were truncated at 30 for data visibility. The actual count was around 110.

Nohara J, Evangelous T, Berry M, Beck W, Mudrak S, Jha S, Reeves RK, Wiehe KJ, Pollara J, Tomaras GD, Bradley T, Ferrari G. Increased Chemokine Production is a Hallmark of Rhesus Macaque Natural Killer Cells Mediating Robust Anti-HIV Envelope Specific Antibody-Dependent Cell-Mediated Cytotoxicity. *Pathogens and Immunity*. 2024;10(1):49–79. doi: 10.20411/pai.v10i1.734

PATHOGENS AND IMMUNITY
IN ASSOCIATION WITH CASE WESTERN RESERVE UNIVERSITY AND THE RICHARD J. FASENMYER FOUNDATION

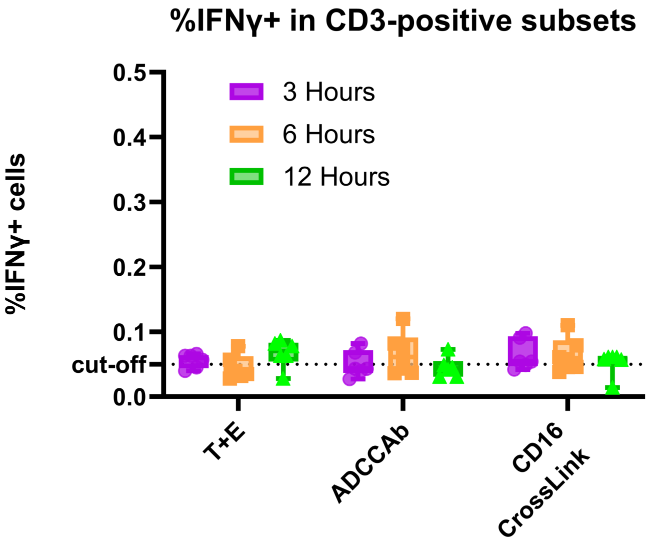

Supplementary Figure 8. IFNγ expression by Non-NK CD3 positive cells over 12-hour incubation. T+E=Target and PBMCs only; ADCCAb= Target cells and PBMCs incubated in the presence of ADCC-mediating antibodies; CD16 Crosslink= Opsonization of RhNK cells by mouse anti-Human CD16 antibodies followed by crosslinking with secondary goat anti-mouse F(ab)'2.

Cut-offs for positive response were set at 0.05%

Nohara J, Evangelous T, Berry M, Beck W, Mudrak S, Jha S, Reeves RK, Wiehe KJ, Pollara J, Tomaras GD, Bradley T, Ferrari G. Increased Chemokine Production is a Hallmark of Rhesus Macaque Natural Killer Cells Mediating Robust Anti-HIV Envelope Specific Antibody-Dependent Cell-Mediated Cytotoxicity. *Pathogens and Immunity*. 2024;10(1):49–79. doi: 10.20411/pai.v10i1.734
