# Supplementary material for: Increased Chemokine Production is a Hallmark of Rhesus Macaque Natural Killer Cells Mediating Robust Anti-HIV Envelope-Specific Antibody-Dependent Cell-Mediated Cytotoxicity
Source: Pathog Immun. 2025 Jan 23;10(1):49–79. doi: 10.20411/pai.v10i1.734 (PMC11792536; doi:10.20411/pai.v10i1.734)
Supplement: Supplementary Tables 1,2,4,5 [file pai-10-049-s02.pdf]

PATHOGENS AND IMMUNITY
In Association with Case Western Reserve University and The Richard J. Fasenmyer Foundation

Supplementary Table 1

Animal used for single-cell RNA sequencing

						CD107a Negaive NK cells			CD107a Positive NK cells		
NHPID	Age(year)	Sex	Weight(kg)	%.NK.cells	Average ADCC(%Gzmb Activity)	Estimated number of cell sequenced	Mean reads per Cell	Median genes per cell	Estimated number of cell sequenced	Mean reads per Cell	Median genes per cell
KP85	6.17	Male	9	2.53	21.85	5,222	93,624	872	1,730	272,611	1,531
LB62	5.23	Male	9.05	1.32	21.3	9,791	58,883	1,399	4,872	116,648	1,655
LC38	5.2	Male	9.25	4.55	19.95	9,781	39,010	1,430	10,780	46,734	1,665
LD26	5.18	Male	9.15	2.6	19.05	6,086	97,461	1,377	6,251	78,010	1,598
LD54	5.16	Male	10.1	2.47	21	6,937	88,293	1,474	6,729	90,953	1,916
LI18	4.16	Male	8.3	1.75	22.15	5,967	95,277	1,462	8,498	63,500	1,330

Animal used for chemokine intracellular staining and luminex assay

NHPID	e (Years, Round	Sex	Weight (kg)
LF37	6	Male	13.80
LF69	6	Male	12.65
LN06	5	Male	11.18
MB72	3	Male	5.40
LP15	4	Male	6.35
LT73	4	Male	7.40
MA99	3	Male	4.46
MC05	3	Male	5.40
LI22	4	Male	8.9
LB62	5	Male	9.05
LF69	5	Male	9.55
LA82	5	Male	11.6
LA71	5	Male	9.7

Nohara J, Evangelous T, Berry M, Beck W, Mudrak S, Jha S, Reeves RK, Wiehe KJ, Pollara J, Tomaras GD, Bradley T, Ferrari G. Increased Chemokine Production is a Hallmark of Rhesus Macaque Natural Killer Cells Mediating Robust Anti-HIV Envelope Specific Antibody-Dependent Cell-Mediated Cytotoxicity. *Pathogens and Immunity*. 2024;10(1):49–79. doi: 10.20411/pai.v10i1.734

PATHOGENS AND IMMUNITY
In Association with Case Western Reserve University and The Richard J. Fasenmyer Foundation

Supplementary Table 2

	p_val	avg_log2FC	pct. In CD107a + cells	pct. In CD107a - cells	p_val_adj
RRAD	0	4.01535	0.523	0.073	0
CRTAM	0	3.376108	0.777	0.184	0
KLF10	0	3.262354	0.552	0.083	0
XCL1	0	3.123023	0.848	0.231	0
RGS16	0	2.885541	0.771	0.24	0
RGCC	0	2.571159	0.868	0.462	0
EGR2	0	2.506592	0.591	0.108	0
TAGAP	0	2.362147	0.747	0.243	0
BCL2A1	0	2.293255	0.564	0.178	0
VSIR	0	2.172465	0.558	0.164	0
REL	0	2.078621	0.705	0.301	0
CCL4L1	0	1.827861	0.914	0.75	0
PARP6	0	1.814666	0.627	0.269	0
TNFSF14	0	1.796029	0.622	0.264	0
NFKB1	0	1.764693	0.633	0.28	0
RNF19A	0	1.751553	0.616	0.297	0
HSD17B4	1.29E-295	1.733357	0.546	0.325	2.03E-291
CCL3	0	1.719627	0.719	0.387	0
GADD45B	4.21E-227	1.696785	0.568	0.38	6.62E-223
CHD4	0	1.680526	0.67	0.341	0
CFLAR	0	1.623127	0.627	0.304	0
MAP2K3	0	1.61732	0.654	0.318	0
HMGA1	0	1.578608	0.534	0.265	0
SERTAD1	0	1.554248	0.598	0.264	0
SRPRB	0	1.548133	0.519	0.25	0
CD69	0	1.533668	0.808	0.509	0
GNL3	4.57E-302	1.464696	0.503	0.244	7.18E-298
SPAG9	0	1.455552	0.764	0.46	0
ZFP36L1	0	1.435016	0.779	0.542	0
TNIP2	1.54E-296	1.426244	0.517	0.256	2.42E-292
LDHA	0	1.418954	0.813	0.591	0
PLEK	0	1.402996	0.834	0.629	0
PARK7	0	1.39017	0.717	0.43	0
HM13	0	1.383583	0.688	0.424	0
FAM3C	1.75E-277	1.358494	0.504	0.247	2.75E-273
HSPA5	0	1.338075	0.925	0.791	0
HSPA9	0	1.27204	0.613	0.345	0
NFKBIA	0	1.265348	0.79	0.486	0
RHOG	0	1.26494	0.756	0.508	0
UFM1	2.55E-237	1.244928	0.512	0.284	4.01E-233
HAX1	2.33E-297	1.20621	0.601	0.343	3.66E-293
ARF4	0	1.203462	0.74	0.486	0
EDF1	0	1.192084	0.895	0.744	0
FOXP1	2.61E-255	1.187237	0.548	0.306	4.09E-251
NFKBIB	3.41E-248	1.147107	0.534	0.288	5.36E-244
FYN	0	1.141773	0.725	0.462	0
ATP1B3	2.85E-261	1.125593	0.572	0.317	4.47E-257

Nohara J, Evangelous T, Berry M, Beck W, Mudrak S, Jha S, Reeves RK, Wiehe KJ, Pollara J, Tomaras GD, Bradley T, Ferrari G. Increased Chemokine Production is a Hallmark of Rhesus Macaque Natural Killer Cells Mediating Robust Anti-HIV Envelope Specific Antibody-Dependent Cell-Mediated Cytotoxicity. *Pathogens and Immunity*. 2024;10(1):49–79. doi: 10.20411/pai.v10i1.734

TIGAR	1.40E-187	1.123982	0.569	0.375	2.20E-183
CYCS	5.56E-235	1.115503	0.571	0.343	8.74E-231
PTPN1	5.90E-254	1.112591	0.605	0.374	9.27E-250
SEC61G	0	1.1045	0.836	0.698	0
KIFBP	0	1.095475	0.732	0.512	0
RAB8B	0	1.091604	0.806	0.599	0
SSR1	2.82E-267	1.090065	0.63	0.399	4.42E-263
PPP4C	0	1.071616	0.758	0.554	0
SEC61B	0	1.068256	0.931	0.835	0
ERGIC2	1.32E-198	1.06325	0.502	0.283	2.08E-194
TGIF1	4.04E-180	1.0397	0.503	0.299	6.34E-176
LMAN1	5.49E-192	1.037868	0.578	0.387	8.62E-188
CD53	0	1.026689	0.8	0.603	0
RASSF5	6.81E-271	1.022983	0.68	0.46	1.07E-266
IRF1	7.01E-247	-1.00717	0.398	0.62	1.10E-242
IKZF1	9.66E-224	-1.0179	0.287	0.515	1.52E-219
TENT5C	3.87E-224	-1.03586	0.266	0.5	6.08E-220
ETS1	0	-1.06949	0.653	0.876	0
HMGB2	3.66E-228	-1.0767	0.318	0.544	5.75E-224
ETFB	1.92E-220	-1.08089	0.32	0.54	3.01E-216
RASSF1	7.72E-219	-1.09341	0.304	0.521	1.21E-214
TAGLN2	0	-1.10722	0.409	0.716	0
CBX3	0	-1.12905	0.464	0.734	0
LNPEP	1.02E-273	-1.16392	0.311	0.551	1.60E-269
SYTL2	0	-1.17835	0.389	0.641	0
GIMAP7	0	-1.18418	0.408	0.693	0
PLAAT3	0	-1.20782	0.39	0.666	0
VIM	0	-1.24539	0.555	0.79	0
MGST3	3.73E-291	-1.27068	0.252	0.515	5.85E-287
PIK3R1	0	-1.27782	0.356	0.694	0
CD2	0	-1.28501	0.337	0.647	0
CORO1A	0	-1.30991	0.502	0.841	0
NTAN1	0	-1.3389	0.346	0.676	0
MFNG	0	-1.34198	0.263	0.526	0
FYB1	0	-1.342	0.319	0.59	0
CRIP1	0	-1.41766	0.568	0.861	0
TMEM14B	0	-1.47606	0.618	0.888	0
S100A4	0	-1.50012	0.408	0.699	0
RIPOR2	0	-1.53555	0.239	0.533	0
TESC	0	-1.58294	0.354	0.696	0
ARHGDIB	0	-1.65762	0.574	0.917	0
YPEL3	0	-1.66236	0.231	0.565	0
IDH2	0	-1.69526	0.213	0.515	0
EZR	0	-1.72069	0.245	0.582	0
EPB41	0	-2.18736	0.178	0.576	0
CXCR4	0	-2.39897	0.124	0.546	0

Nohara J, Evangelous T, Berry M, Beck W, Mudrak S, Jha S, Reeves RK, Wiehe KJ, Pollara J, Tomaras GD, Bradley T, Ferrari G. Increased Chemokine Production is a Hallmark of Rhesus Macaque Natural Killer Cells Mediating Robust Anti-HIV Envelope Specific Antibody-Dependent Cell-Mediated Cytotoxicity. *Pathogens and Immunity*. 2024;10(1):49–79. doi: 10.20411/pai.v10i1.734

PATHOGENS AND IMMUNITY
In Association with Case Western Reserve University and The Richard J. Fasenmyer Foundation

Supplementary Table 4

Chemokines(n=5)	T+E (pg/mL)	ADCCAbs 6Hr (pg/mL)	ADCCAbs 24Hr (pg/mL)
CCL3(MIP-1α)	35.75	57.65	77.32
CCL4(MIP-1β)	296.09	553.92	894.47
CCL5(RANTES)	183.36	129.47	86.95

Supplementary Table 5

Target	Clone	Fluorophore	Vendor	Cat#
CD107a	H4A3	PE-Cy5	BD Biosciences	555802
Viability stain		BV510	ThermoFisher Scientific	L34957
CD8	SK1	APC-H7	BD Biosciences	560179
NKG2A/C	Z199	PE	Beckman-Coulter	IM3291
CD16	3G8	PE-CF594	BD Biosciences	562293
CD14	M5E2	BV650	BD Biosciences	563420
CD56	NCAM16.2	PE-CY7	BD Biosciences	335809
CD3	SP34.2	PacBlue	BD Biosciences	558124
CCL4	D21-1351	R718	BD Biosciences	567090
IFNγ	B27	APC	BD Biosciences	502512
CD20	2H7	Alexa700	BD Biosciences	560631

Nohara J, Evangelous T, Berry M, Beck W, Mudrak S, Jha S, Reeves RK, Wiehe KJ, Pollara J, Tomaras GD, Bradley T, Ferrari G. Increased Chemokine Production is a Hallmark of Rhesus Macaque Natural Killer Cells Mediating Robust Anti-HIV Envelope Specific Antibody-Dependent Cell-Mediated Cytotoxicity. *Pathogens and Immunity*. 2024;10(1):49–79. doi: 10.20411/pai.v10i1.734
